# Familial Combined Hyperlipidemia: Myth or Reality?

**DOI:** 10.1007/s11883-025-01289-9

**Published:** 2025-04-01

**Authors:** M. C. G. J. Brouwers, B. Klop, J. Ribalta, M. Castro Cabezas

**Affiliations:** 1 Department of Internal Medicine, Division of Endocrinology and Metabolic Disease, Maastricht UMC+, Maastricht, the Netherlands; 2 Cardiovascular Research Institute Maastricht (CARIM), Maastricht University, Maastricht, the Netherlands; 3 Department of Cardiology, Anna Hospital, Geldrop, the Netherlands; 4 Facultat de Medicina I Ciències de La Salut, Unitat de Recerca en Lípids I Arteriosclerosi, Universitat Rovira I Virgili, Reus, Spain; 5 Institut d’Investigació Sanitària Père Virgili, Reus, Spain; 6 Centro de Investigación Biomédica en Red de Diabetes y Enfermedades Metabólicas Asociadas, Madrid, Spain; 7 Department of Internal Medicine, Franciscus Gasthuis & Vlietland, Rotterdam, the Netherlands; 8 Department of Internal Medicine, Division of Endocrinology, Erasmus University Medical Center, Rotterdam, the Netherlands; 9 Julius Clinical, Zeist, the Netherlands

**Keywords:** Free fatty acids, Triglycerides, Hyperlipidemia, Fatty liver disease, VLDL overproduction

## Abstract

**Purpose of Review:**

Familial combined hyperlipidemia (FCHL) was first described by Goldstein and co-workers in 1973 as a multiple-type hyperlipidemia in pedigrees with premature myocardial infarction. However, it can be questioned what actually defines FCHL.

**Recent Findings:**

Although initially regarded as an autosomal dominant disorder, quantitative trait linkage analyses have revealed multiple genes that are associated with the FCHL phenotype. With the advent of genome-wide association studies and next generation sequencing it has been confirmed that FCHL is a polygenic disorder and the associated gene variants, mostly with a triglyceride-raising effect, are not unique to FCHL. Furthermore, epidemiological studies have demonstrated that the multiple-type hyperlipidemia is also not specifically confined to FCHL.

**Summary:**

This review provides a historical overview of the metabolic and genetic abnormalities that characterize FCHL. Integration of these findings with recent population-based, genetic studies results in a new pathophysiological concept of FCHL. This model provides practical guidance on how to approach an individual patient with an ‘FCHL phenotype’.

## Introduction

Familial combined hyperlipidemia (FCHL) was described for the first time by Goldstein and co-workers in Seattle [1]. In that same year in 1973, Rose and coworkers [2] and Nikkilä and Aro [3] published similar papers describing the same condition. Goldstein et al. [1] suggested that FCHL was the most frequent dominantly inherited disorder of lipid metabolism leading to increased risk of atherosclerosis which eventually was confirmed by others many decades later [4, 5]. All these investigators published extensive pedigree analyses clearly suggesting that this was a dominantly inherited condition. Most investigators and clinicians have used clinical criteria like “multiple type hyperlipidemia” [1–5] in relatives of index patients, increased concentrations of plasma apolipoprotein (apo) B and a positive family history of premature cardiovascular disease (CVD) to establish the diagnosis of FCHL.

In search for the genetic basis of FCHL, different groups started the quest to find the single gene associated to this disorder, without any success so far. Nowadays, it is well accepted that FCHL is a polygenic disorder in which different mutations can lead to the same phenotype [6]. Nowadays there is also indirect evidence for a modest contribution of rare variants with variable penetrance or a combination of common variants leading to the more generalized phenotype of combined hyperlipidemia or multiple type hyperlipidemia [7]. In FCHL different lipid phenotypes can exist within one pedigree and these patients are prone to develop cardiovascular complications. They also have an increased risk for type 2 diabetes and steatotic liver disease [8–10].

This review provides a historic overview of FCHL including its metabolic characterization, the genetic basis and why FCHL remains a valid and useful diagnosis.

## Metabolic Characterization of Familial Combined Hyperlipidemia

Several metabolic characteristics of FCHL have been described that seem to be rather common in these subjects (Table 1). One of the best studied and earliest identified metabolic abnormalities in FCHL is hepatic VLDL overproduction [11–13]. In addition, and possibly closely related, insulin resistance is also a distinctive characteristic in FCHL [14–16]. Other metabolic disturbances, most likely as a consequence of the hepatic VLDL overproduction are delayed clearance of chylomicron remnants [17–19] and high concentrations of marginated atherogenic lipoproteins most likely bound to the endothelium [20]. Some experts have suggested that the primary defect leading to hepatic VLDL overproduction could be an impaired metabolism of plasma free fatty acids (FFA) [14, 16], by inefficient peripheral cellular uptake resulting in enhanced hepatic flux of these FFA. In one in vivo experiment this concept was validated showing exaggerated production of postprandial ketone bodies in FCHL subjects [21]. This latter study in a small number of subjects is the only one supporting this concept of enhanced hepatic fatty acid flux. The basis for this hypothesis lies in the work by Sniderman and Cianflone showing impaired action of acylation stimulating protein (ASP) in subjects with elevated apolipoprotein (apo) B levels (hyperapoB) [22, 23]. ASP appeared to be identical to C3adesArg, an immunologically inactive split product of the complement component 3 (C3). Several in vivo studies established the connection between triglyceride-rich lipoprotein metabolism [24], the FCHL phenotype and C3, especially in relation to postprandial metabolism and fatty acid handling [20, 25]. In one FCHL family, a mutation in the C5L2 gene (the C3adesArg/ASP-receptor) was considered to contribute to the phenotypic expression supporting the role of the complement system in FCHL expression [26]. Although later studies provided more insight into the role of the complement system activation to lipoprotein transport and metabolism [27], the pathogenetic role in FCHL remains unclear.

**Table 1:** Metabolic characteristics described in patients with familial combined hyperlipidemia

VLDL-overproduction
Elevated fasting apo B
Decreased clearance of chylomicron remnants
Postprandial elevated FFA
Increase in postprandial ketone bodies
Impaired postprandial C3 response
Small dense LDL
Insulin resistance Steatotic liver disease
Decreased ASP action (in vitro)
Enhanced margination of apo B in vivo
Enhanced glucose-dependent leukocyte activation
Insufficient response to standard lipid lowering therapy

apo = apolipoprotein, ASP = acylation stimulating protein, FFA: free fatty acids; LDL = low density lipoprotein, VLDL = very low density lipoprotein

Later work from others showed that the impaired fatty acid handling may be present only in the hypertriglyceridemic FCHL subjects [28, 29]. In addition, in vitro studies by Peter Arner’s group suggested that hormone-sensitive lipase (HSL) activity, measured in isolated fat cells, was decreased in FCHL subjects [30] and also in vitro activation was decreased compared to controls [31]. However, in vivo studies in FCHL subjects evaluating HSL modulation failed to support these findings [32], although insulin resistance at the level of HSL was confirmed.

**Fig. 1:**
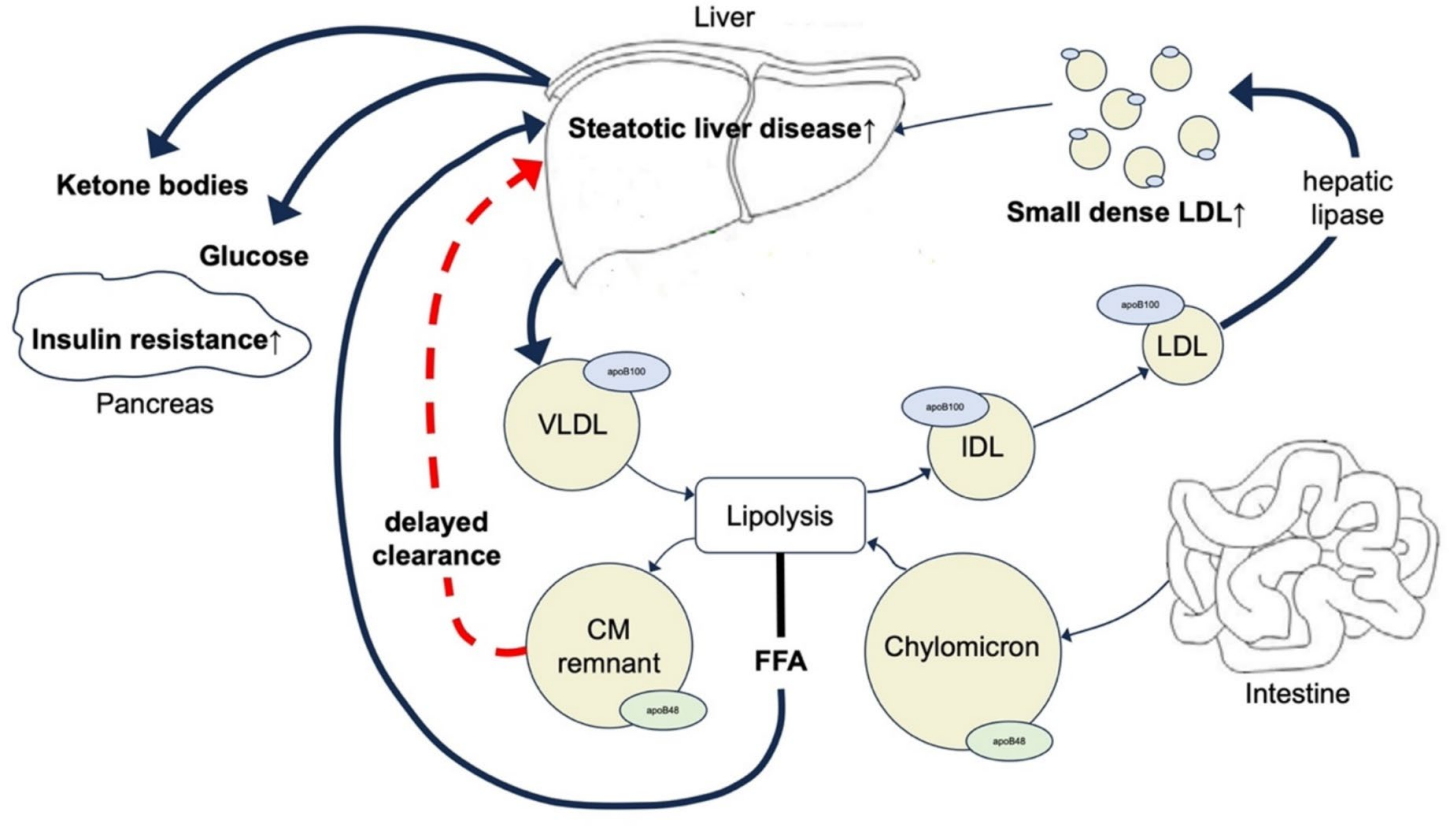
Schematic representation of triglyceride-rich lipoprotein metabolism with emphasis on processes leading to generation of a steatotic liver as suggested in FCHL

Many different metabolic processes influence plasma FFA concentrations (Fig. 1) [33]. For example high plasma VLDL are usually accompanied by higher FFA levels, due to the larger amount of substrate available for extracellular lipolysis. Moreover, plasma FFA concentrations are determined by the balance between intravascular lipolysis of triglycerides by lipoprotein lipase (LPL) and the delivery of these VLDL by blood flow velocity on the one hand and the cellular uptake of FFA on the other hand. In this latter process, several factors play a role: the acylation-stimulating protein (ASP)/C3-pathway, fatty acid transporter/CD36 and other transmembrane transporters [34, 35]. In some patients a decreased LPL activity has been described, which may have contributed to the presenting phenotype. However, in vivo fatty acid kinetics were not reported in those subjects [36, 37].

**Fig. 2:**
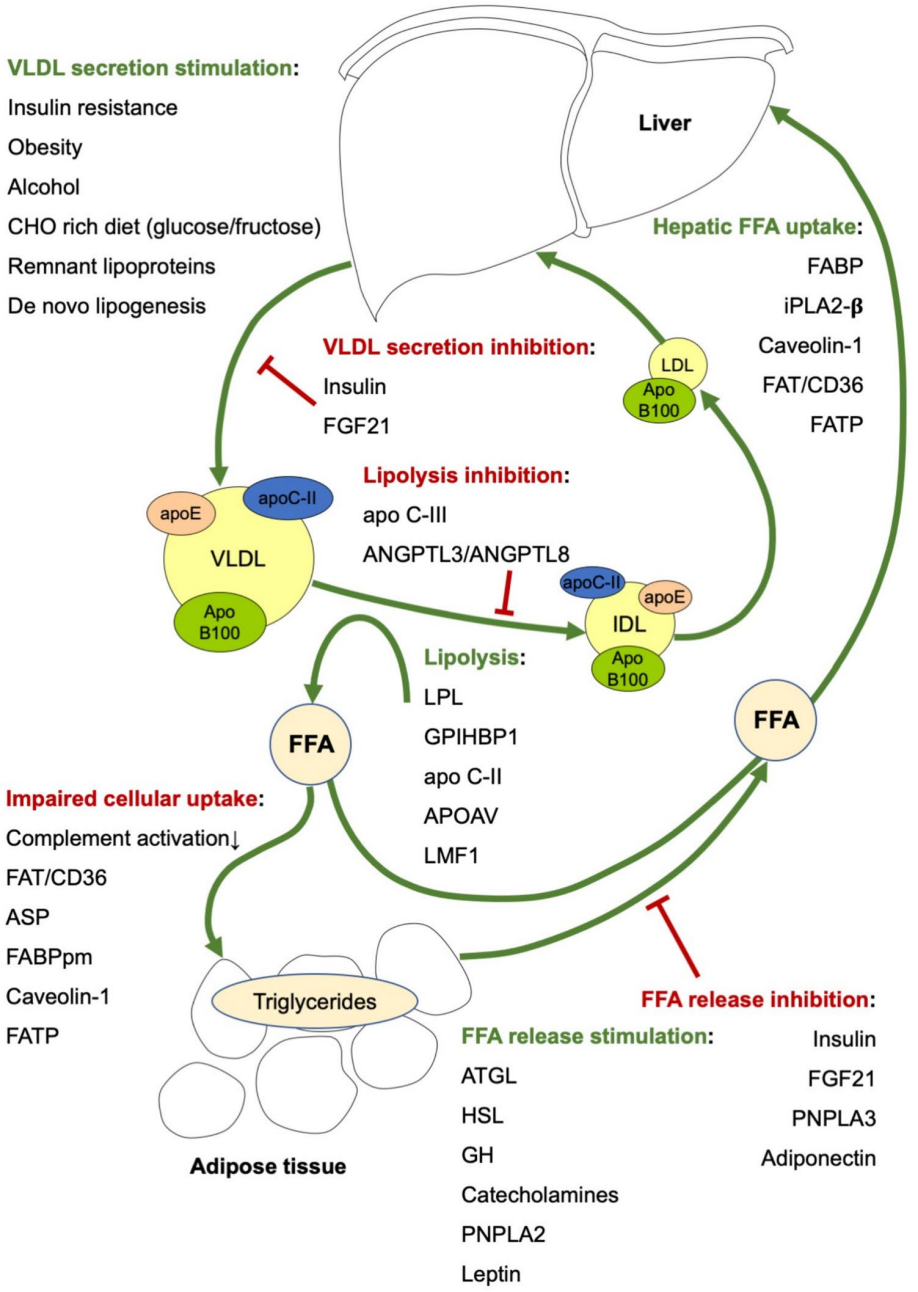
Overview of molecular determinants of FFA metabolism determining cellular uptake, storage and release

FFA metabolism is a complex and not yet fully understood process. Many different molecules play an important role in the generation, cellular uptake and storage of fatty acids (Fig. 2). Mutations in one or several genes involved in fatty acid handling may lead to impaired peripheral fatty acid uptake and therefore, enhanced flux of fatty acids to the liver. Patients with FCHL are characterized by an increased intrahepatic lipid content. Forty-nine percent fulfil the criteria for ultrasound-based steatotic liver disease [8]. The prevalence is even higher (~ 66%) among patients with the hypertriglyceridemic phenotype. Heritability analyses in FCHL pedigrees have shown that 20–36% of the variability in intrahepatic lipid content – using serum alanine aminotransferase levels as proxy – can be attributed to genetic factors [38]. Stable isotope studies in non-FCHL individuals have shown that higher intrahepatic lipid content is associated with VLDL overproduction, which is probably mediated by lipid-induced insulin resistance [39].

It is quite conceivable that different mutations may lead to the same FCHL phenotype. In theory, each family with FCHL may have a different mutation. This would explain why the genome-wide association studies (GWAS) analyses have not resulted in one single gene explaining the phenotype. In our opinion, a specific analysis per pedigree evaluating these fatty acid pathways would shed more light in the metabolic and genetic basis of FCHL.

## Genetic Basis of Familial Combined Hyperlipidemia

Our present understanding can be summarized as follows: many years of research on the genetic basis of FCHL have ruled-out a single gene cause and, conversely, more recent studies benefiting from next-generation methodology have convincingly demonstrated its polygenic nature with a significant contribution of single nucleotide polymorphisms (SNPs) with a triglyceride-raising effect [40].

Upon the initial identification of the first pedigrees back in 1973 [1], it was believed that its phenotypic segregation would fit better with a pattern of autosomal dominant inheritance. This led to decades of investigations in which the search for a major single gene effect was pursued mostly through linkage analyses simultaneously accompanied by association studies identifying loci capable of modulating the FCHL phenotype. The first study reporting data suggestive of a significant link between a genomic region in the APOA1-C3-A4 gene cluster and seven FCHL families was published in 1991 by Wojciechowski et al. [41]. Although this result could not be consistently replicated, it pointed out a major modulatory region for some of the main FCHL metabolic characteristics such as increased number of apo B containing lipoproteins [12] or delayed postprandial lipemia and increased FFA levels [42]. The identification by Pennacchio et al. [43] of a fourth member of the cluster with a very strong modulatory role on triglycerides, APOA5 [43, 44], further increased the interest of this region in relation to FCHL. Carriers of the −1131 T rare variant predisposing to hypertriglyceridemia were over-represented among FCHL patients [45]. Subsequently, a large study on 128 kindreds which analyzed the entire APOA1-C3-A4-A5 region, concluded by combining linkage and association studies that this region was a significant contributor to the FCHL phenotype among families in Northern Europe [46]. In trying to find a causal gene for FCHL, a similar promising finding was made in 1998. Two groups simultaneously identified a novel locus 1q21-23 linked to FCHL in 31 extended Finnish families [47] and their syntenic region in the mouse genome which was associated with combined hyperlipidemia and elevated levels of apo B-100 [48]. Subsequent studies in large Finnish and Dutch FCHL pedigrees identified Upstream stimulating factor 1 (USF-1), a transcription factor involved in the regulation of lipid, glucose and adipose tissue metabolism, as the most likely candidate for the association between this genomic region and FCHL [49, 50]. These are two examples of linkage studies pointing at genomic regions that predispose to the FCHL phenotype in certain families but that could not be generally labelled as FCHL-causative. In this context, the absence of studies identifying a single gene or genetic region capable of explaining the FCHL phenotype, together with the growing body of evidence clearly showing that common SNPs were underlying numerous disorders, shifted the general point of view towards a polygenic nature of FCHL.

Later studies supported the polygenic basis of FCHL [51–54]. These studies operated with polygenic risk scores (PRS), which incorporated SNPs drawn from GWAS showing to significantly modify a given trait. Based on GWAS data, a PRS is built by incorporating a variable number of SNPs that modulate a trait or a phenotype in the same direction [55]. While most of these GWAS analyses were not specifically aimed at FCHL as they had a wider scope on lipids, dyslipidemia or cardiovascular risk [56], some did. Rippatti et al. imputed nine million variants to 234 FCHL individuals from 53 Finnish families [52]. Trinder et al. explored 349,222 unrelated participants of European ancestry in the UK Biobank in search of FCHL individuals using 5 different selection criteria for FCHL [51] and Gill et al. followed a similar strategy in 259 individuals with combined hyperlipidemia [53]. Although the results of these studies may vary slightly due to the clinical characteristics of the included subjects or to the selection of the SNPs included in the PRS, they coincide in that approximately 25% of FCHL individuals show an over-representation of triglyceride-raising SNPs, whereas the presence of SNPs raising LDL-C seems clearly lower [52, 53]. Of note, since PRS SNPs derive from GWAS, many of them are located within novel genes, intronic or intergenic regions that need to be further studied and which may provide insight into the etiopathogenesis of FCHL.

Although nowadays the polygenic nature seems undisputed, decades of research of single-gene defects based on the study of FCHL kindreds has led to the identification of numerous “FCHL-related genes”. Some of these genes were initially identified by linkage studies among families where those variants showed a very significant impact as in the already mentioned cases of USF1 [47, 50] and the A1-C3-A4-A5 gene cluster [41]. A plethora of genes and genetic variants have been reported to play a modulatory role of the main characteristics of the FCHL phenotype [56] but in most cases they are not present in FCHL in a higher proportion than in hypertriglyceridemia [53]. Some examples of these genes might be: genes involved in triglyceride metabolism (LPL, APOC3, APOA5, LIPC, CETP or GPIHBP1) [42, 45, 56, 57]; fat accumulation and VLDL overproduction (GCKR) [58], dysfunctional adipose tissue (USF-1) or LDL metabolism (LDL-R, PCSK9 and SREBP-2) [53, 56]. An intriguing candidate gene is ANGPTL3 whose loss of function causes the exact opposite phenotype, familial combined hypolipidemia. However, its implication in FCHL has not been confirmed [59].

Two facts have been convincingly demonstrated in relation to the genetic basis of FCHL, its polygenic nature and the over-representation of triglyceride-rising SNPs. However, there still remains a large part of the underlying predisposition to be unraveled. A more detailed definition of the clinical phenotypes and the incorporation of all forms of gene-environmental interactions into the equation will probably help better understand the FCHL phenotype to establish more firmly FCHL as a relevant clinical entity.

## The Multiple-type Hyperlipidemia

One of the most characteristic features of FCHL is the presence of different lipoprotein phenotypes at one timepoint in relatives [1, 2, 5], the “multiple type hyperlipidemia”. In addition, it was also reported that an individual patient may show different phenotypes during a longer period of time and the term “multiple type hyperlipidemia” was also used for this clinical feature [60].

One of the few studies trying to describe the variability of lipoprotein phenotypes in FCHL came from the Utrecht lipid group challenging this concept [61]. In this study 18 FCHL patients and 16 matched controls with multiple lipid and lipoprotein measurements, no significant differences in fasting nor postprandial lipid profile variability were observed between the groups. In this respect, others had already shown in non-FCHL subjects that fasting plasma lipids are widely variable [62].

The Maastricht group subsequently studied the relationship between serum triglycerides and cholesterol to disentangle the conundrum of the multiple-type hyperlipidemia. Based on the premise that triglyceride-rich VLDL1 particles – which are abundant when serum triglycerides > 1.5 mmol/L – exchange their triglycerides for cholesteryl esters from LDL particles [63], they hypothesized a parabolic relationship between serum triglycerides and LDL-C. Indeed, such a relationship was observed in two FCHL cohorts [64]. Again, the parabolic relationship was not unique for FCHL, it was also observed in the general population and in subjects with type 2 diabetes mellitus [65].

**Fig. 3:**
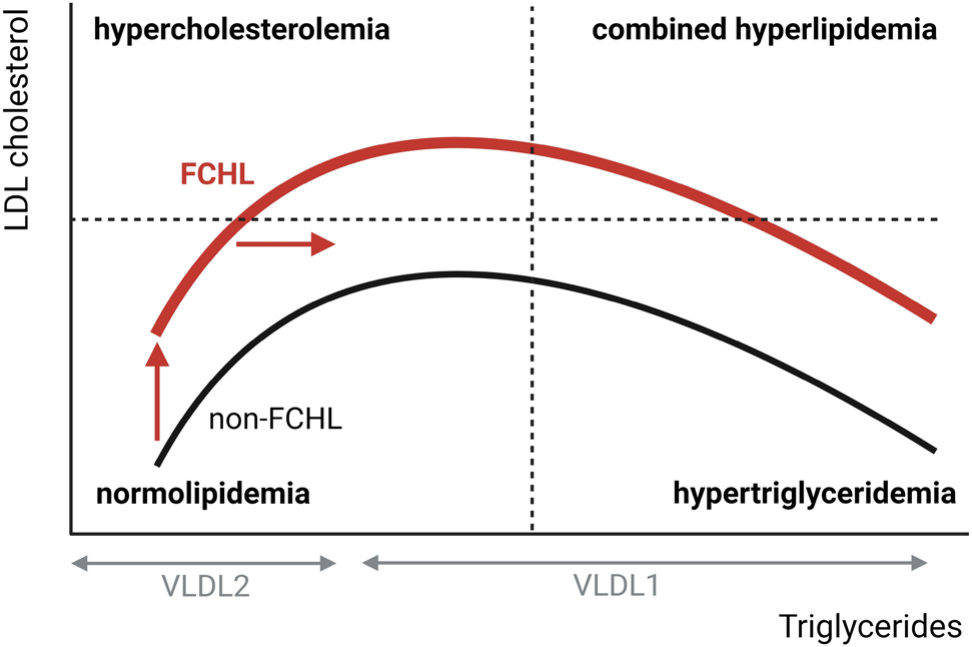
Conceptual model of the multiple-type hyperlipidemia in FCHL. When serum triglycerides are low (< 1.5 mmol/L) VLDL2 particles predominate and are particularly catabolized into LDL particles. When serum triglycerides increase (> 1.5 mmol/L) triglyceride-rich VLDL1 particles become abundant and exchange their triglycerides for cholesteryl esters from LDL particles. This explains the inverse relationship between serum triglycerides and LDL cholesterol at higher serum triglycerides levels. In FCHL, individuals are genetically predisposed to higher triglycerides (horizontal red arrow). Furthermore, a similar genetic predisposition causes a shift of the parabolic curve upwards (vertical red arrow). As consequence, any change in serum triglycerides – caused by a change in intrahepatic lipid accumulation – will cause a change in the lipid phenotype. Figure created with Biorender.com

So, how to reconcile these non-FCHL specific findings to a supposedly FCHL specific phenomenon? By integrating the hitherto described metabolic and genetic defects that have been observed in FCHL, the following model can be proposed to account for the multiple-type hyperlipidemia in FCHL (Fig. 3). A genetic predisposition drives progression to hypertriglyceridemia in individuals with FCHL. Similarly, a genetic predisposition to high cholesterol levels may shift the parabolic curve upwards resulting in an ideal position for the cut-off for hypercholesterolemia. As a consequence, an individual with FCHL can display all types of hyperlipidemia, i.e. hypercholesterolemia, hypertriglyceridemia and combined hyperlipidemia, depending on the actual levels of serum triglycerides. Longitudinal studies have shown that the variability in the hypertriglyceridemic phenotype in FCHL is associated with body mass index, insulin resistance and intrahepatic lipid content [66–68]. Hence, any change in intrahepatic lipid content – caused by amongst others a change in body weight – affects insulin-mediated VLDL production and, as such, the position in the parabolic curve (Fig. 3). Of interest, intrahepatic lipid content appears highly variable as it can already be modulated by a single high-fat meal [69]. Therefore, establishing the diagnosis of FCHL in clinical practice can be challenging due to the potential presence of a multiple-type hyperlipidemia over time.

## FCHL: Myth or Reality?

Notwithstanding the metabolic and genetic overlap with other entities, fact is that clinicians regularly see patients with combined hyperlipidemia and relatively normal LDL-C concentrations who develop cardiovascular complications at a relatively young age. These subjects have sometimes mildly to moderately elevated plasma triglycerides, elevated plasma apo B concentrations and a positive family history of CVD. This phenotype is not at all infrequent in clinical practice. When evaluating more closely the presenting phenotype and the relatives of the index subject, different lipid phenotypes may be found in one pedigree. More importantly, a recent 15-year follow-up study has shown that patients with FCHL are still prone to develop cardiovascular complications (HR: 5.4; 95%CI: 2.0–14.6) – even in the current era of aggressive lipid lowering therapy – and that this increased risk is not adequately captured by cardiovascular risk charts, such as SCORE [70]. Follow-up on incident type 2 diabetes mellitus in the same cohort also revealed a 6.3 greater risk (95%CI: 2.4–16.8) in FCHL, which was largely explained by the presence of steatotic liver disease, as assessed by ultrasound (HR: 6.9; 95%CI: 1.1–42.2) [10].

**Fig. 4:**
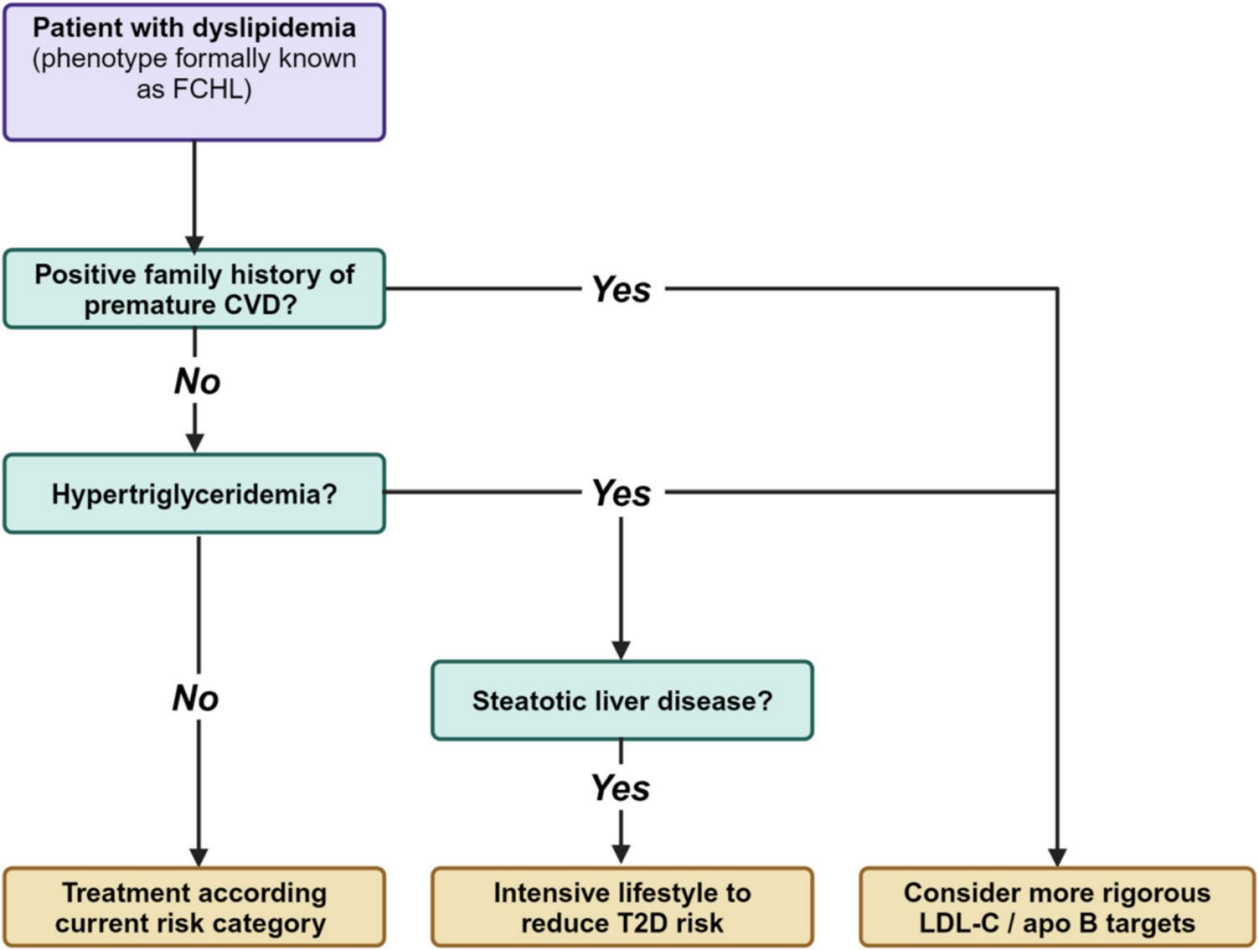
Clinical diagnostic and treatment approach to patients with the FCHL phenotype

So, if one would decide to abandon FCHL as a distinct familial disorder, there would be an even greater risk that patients with the ‘FCHL-phenotype’ and their relatives are not adequately identified with regard to their cardiometabolic risk. Any alternative diagnosis should adequately capture this risk. The current clinical guidelines may provide some guidance as summarized in Fig. 4.

The first description of FCHL started in pedigrees with premature myocardial infarction [1]. Based on this selection it is conceivable that the increased cardiovascular risk in these pedigrees was not solely conferred by dyslipidemia. Other factors, both established and yet unknown, may have contributed as well. Hence, it is important to take into account a positive family history of premature cardiovascular disease when treatment targets are defined in an individual patient with dyslipidemia [71]. Second, it is well-known that LDL cholesterol levels underestimate cardiovascular risk in individuals with hypertriglyceridemia, due to the presence of atherogenic remnant and small-dense LDL particles. Non-HDL cholesterol or (preferably) apo B levels should be measured and used as a (secondary) treatment target [71]. Finally, the increased risk of type 2 diabetes mellitus should be taken into account in a patient with dyslipidemia, particularly in individuals with hypertriglyceridemia. Assessment of intrahepatic lipid accumulation (by either ultrasound or MRI) may aid in risk stratification. It is anticipated that individuals with steatotic liver disease will mostly benefit from intensive lifestyle interventions in order to reduce serum lipid levels and reduce the risk of type 2 diabetes mellitus.

In conclusion, should FCHL be considered a reality or a myth? It appears that modern genetic technology has caught up with the concept of FCHL, that was defined more than five decades ago. FCHL can now be viewed as a ‘bag of susceptibility genes’ – homogeneous within a pedigree but heterogeneous between pedigrees – that gives rise to a lipid phenotype when there is interaction with environmental factors. Although this new view demands a change in taxonomy, it should be emphasized that this pathophysiological principle also holds true for many other metabolic disorders, such as obesity, MASLD and type 2 diabetes mellitus. These entities – by many regarded as homogeneous disorders – are also polygenic in nature. Of interest, data-driven approaches have recently identified different type 2 diabetes mellitus and MASLD subtypes that are differentially related with cardiometabolic outcomes [72–75]. Such an approach may also be beneficial for polygenic lipid disorders. Pending clinical application, it is imperative that patients with the ‘FCHL phenotype’ are aggressively treated in order to prevent cardiometabolic complications.

## Conclusions

We strongly believe that there is sufficient evidence to use the FCHL diagnosis in clinical practice to identify patients at high cardiovascular risk. FCHL is not a monogenic disorder but rather a polygenic disease mainly affecting triglyceride metabolism significantly increasing cardiovascular risk and very closely associated to insulin resistance and disturbances in fatty acid metabolism.

## Key References

- He Q, Chen Y, Wang Z, He H, Yu P. Cellular Uptake, Metabolism and Sensing of Long-Chain Fatty Acids. Front Biosci (Landmark Ed). 2023 Jan 16;28(1):10.
  - This extensive review describes the cellular uptake, metabolism and sensing of long-chain fatty acids in extensive detail.
- Gill PK, Hegele RA. Familial combined hyperlipidemia is a polygenic trait. Curr Opin Lipidol. 2022 Apr 1;33(2):126–32.
  - This is an up to date review on the polygenic trait as basis for familial combined hyperlipidemia.
- Trinder M, Vikulova D, Pimstone S, Mancini GBJ, Brunham LR. Polygenic architecture and cardiovascular risk of familial combined hyperlipidemia. Atherosclerosis. 2022 Jan;340:35–43.
  - This is a large case–control GWAS (N = 349,222), which identified 175 independent loci associated with FCHL. Risk on incident coronary artery disease was similar between subjects with either FCHL or with monogenic familial hypercholesterolemia.
- Gill PK, Dron JS, Berberich AJ, Wang J, McIntyre AD, Cao H, et al. Combined hyperlipidemia is genetically similar to isolated hypertriglyceridemia. J Clin Lipidol. 2021;15(1):79–87.
  - A cohort study, which showed that combined hyperlipidemia was genetically similar to isolated hypertiglyceridemia. Increased LDL-C in combined hyperlipidemia were not associated with common or rare LDL-C related genetic variants.
- Bea AM, Franco-Marín E, Marco-Benedí V, Jarauta E, Gracia-Rubio I, Cenarro A, et al. ANGPTL3 gene variants in subjects with familial combined hyperlipidemia. Sci Rep. 2021 Mar 26;11(1):7002.
  - A case–control study, which identified a 2.7 fold increase in four variants of ANGPTL3 in a large group of unrelated subjects with FCHL, but no gain of function mutations in ANGPTL3.
- Jamialahmadi O, De Vincentis A, Tavaglione F, Malvestiti F, Li-Gao R, Mancina RM, et al. Partitioned polygenic risk scores identify distinct types of metabolic dysfunction-associated steatotic liver disease. Nat Med. 2024 Dec;30(12):3614–23.
  - This study which identified 27 previously unknown genetic loci associated with MASLD. This resulted in a polygenic risk score suggesting the presence of two distinct types of MASLD with either a more aggressive liver disease or a higher risk of cardiometabolic disease.

## Data Availability

No datasets were generated or analysed during the current study.
